# Presence of Middle Cerebellar Peduncle Sign in *FMR1* Premutation Carriers Without Tremor and Ataxia

**DOI:** 10.3389/fneur.2018.00695

**Published:** 2018-08-22

**Authors:** Jessica L. Famula, Forrest McKenzie, Yingratana A. McLennan, James Grigsby, Flora Tassone, David Hessl, Susan M. Rivera, Veronica Martinez-Cerdeno, Randi J. Hagerman

**Affiliations:** ^1^MIND Institute, University of California Davis Medical Center, Sacramento, CA, United States; ^2^Department of Psychiatry and Behavioral Sciences, University of California Davis School of Medicine, Sacramento, CA, United States; ^3^School of Medicine, University of Colorado, Denver, CO, United States; ^4^Department of Biochemistry and Molecular Medicine, University of California Davis School of Medicine, Sacramento, CA, United States; ^5^Department of Psychology, University of California Davis, Davis, CA, United States; ^6^Department of Pathology and Laboratory Medicine, Sacramento, CA, United States; ^7^Institute for Pediatric Regenerative Medicine and Shriners Hospitals for Children Northern California, Sacramento, CA, United States; ^8^Department of Pediatrics, University of California Davis School of Medicine, Sacramento, CA, United States

**Keywords:** FXTAS, MCP sign, movement disorder, MRI, *FMR1* premutation

## Abstract

Here we report five cases of male *FMR1* premutation carriers who present without clinical symptoms of the fragile X-associated tremor/ataxia syndrome (FXTAS), but who on MRI demonstrate white matter hyperintensities in the middle cerebellar peduncles (MCP sign) and other brain regions, a rare finding. MCP sign is the major radiological feature of FXTAS; it is therefore remarkable to identify five cases in which this MRI finding is present in the absence of tremor and ataxia, the major clinical features of FXTAS. Subjects underwent a detailed neurological evaluation, neuropsychological testing, molecular testing, and MRI evaluation utilizing T2 imaging described here. Additional white matter disease was present in the corpus callosum in four of the five cases. However, all cases were asymptomatic for motor signs of FXTAS.

## Introduction

Fragile X-associated tremor/ataxia syndrome (FXTAS) is a neurodegenerative condition resulting from a premutation (55-200 CGG repeats) in the *FMR1* gene located on the X chromosome (1, 2). Nearly half of all male *FMR1* premutation carriers may develop FXTAS before age 70, with the disease penetrance rising to 75% after age 80 (3, 4). FXTAS can share symptoms with other movement disorders; therefore *FMR1* gene testing clarifies the diagnosis if an intention tremor and/or cerebellar ataxia manifests (5). FXTAS typically presents in those who are >50 years of age. There may be cognitive decline and characteristic radiological findings, including white matter hyperintensities in the middle cerebellar peduncle (MCP sign) in 60% of males and 16% of females with FXTAS (4, 6, 7). The MCP sign represents the major radiological diagnostic criterion for FXTAS and confirms a definite diagnosis of FXTAS when it occurs with tremor and/or ataxia (8). Additional MRI findings include white matter hyperintensities in the periventricular regions and splenium of the corpus callosum (CC) (9, 10).

FXTAS is presumably caused by excessive *FMR1* mRNA that occurs in the premutation, leading to RNA toxicity (11). This includes sequestration of a number of proteins including DROSHA and DGCR8, elevated calcium levels in neurons causing activation of calpain, and a cascade of molecular changes leading to miRNA dysregulation, chronic mitochondrial dysfunction and the formation of FMRpolyG through repeat-associated non-AUG (RAN) translation (2, 12–17). Classic neuropathological findings are eosinophilic intranuclear inclusions that are tau and synuclein negative in neurons, astrocytes, and in Purkinje cells (18, 19).

Here we report five cases of male premutation carriers who present without clinical symptoms of FXTAS but demonstrate the MCP sign, previously seen only in two premutation cases with minimal or no symptoms reported by Loesch et al. (20). The Loesch et al. cases included one individual aged 52 years and another aged 39 years, both showing no clinical symptoms of neurodegeneration, but presenting with significant MCP signs. Given the absence of longitudinal data collection in these cases, it was uncertain how fast FXTAS may develop after the presence of the MCP sign.

## Cases

Case 1 is a 65-year-old, right-handed male with 84 cytosine-guanine-guanine (CGG) repeats, who denied tremor and ataxia. On examination his blood pressure was 177/87 mmHg and his heart rate was 62 bpm. This was consistent with reported history of and treated with metoprolol and candesartan. His body mass index (BMI) was 29.3. On neurological examination, finger-to-nose touching was without tremor and his arm movements were normal. His deep tendon reflexes were 1 to 2+ in the upper extremities, 3+ at the knees, and 2+ at the ankles. His temperature sensation was normal and his vibration sensation was absent in both great toes. Tandem walking was performed normally. No cognitive abnormalities were present on neuropsychological examination and no psychiatric symptoms were reported.

**Figure 1:**
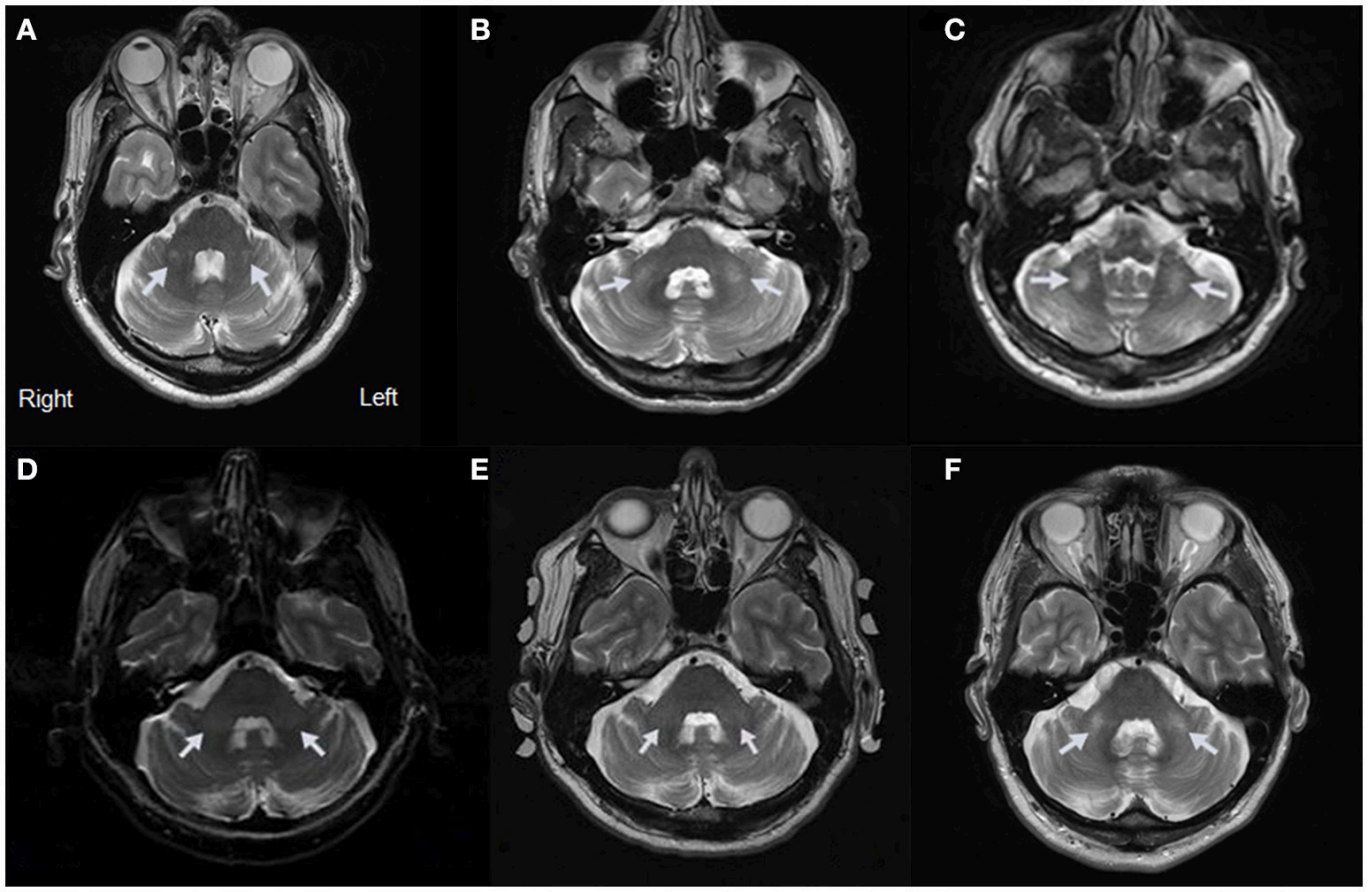
MRI Evidence of MCP Sign for Cases 1–5. **(A)** Axial view of Case 1 showing a hyperintensity of the MCP. **(B)** Axial view of Case 2 showing a hyperintensity of the MCP. **(C)** Axial view of Case 3 showing a hyperintensity of the MCP. **(D)** Axial view of Case 4 showing a hyperintensity of the MCP at age 60. **(E)** Axial view of Case 4 showing a hyperintensity of the MCP at age 64. **(F)** Axial view of Case 5 showing a hyperintensity of the MCP.

His MRI demonstrated the emergence of a faint MCP sign (Figure 1A). His CC was slightly thin with minimal hyperintensity of the splenium of the CC. There was no significant atrophy but there was a hint of white matter hyperintensity in the insula bilaterally.

Case 2 is a 50-year-old, right-handed male carrier with 102 CGG repeats who denied tremor and ataxia. He had a history of multiple concussions from sports injuries in high school and college.

On examination his blood pressure was 147/82 mmHg and his heart rate was 48 bpm. His BMI was 25.8. Finger-to-nose touching was without tremor, and deep tendon reflexes were symmetrical and 1+ at the upper extremities, 2+ at the knees, and 2+ at the ankles. His vibration sense was mildly decreased in the lower extremities. His tandem gait was normal. No cognitive abnormalities were present on neuropsychological examination and no psychiatric symptoms were reported.

He presented with the MCP sign on MRI (Figure 1B). He also had deep cerebellar white matter disease adjacent to the dentate nuclei, white matter hyperintensity in the splenium of the CC, mild volume loss involving the vermis and cerebellar hemispheres, and mesencephalic changes with widened third ventricle. In addition, an indentation in the superior aspect of the CC was thought to relate to a small vascular malformation or aneurysm.

Case 3 is a 62-year-old, right-handed male carrier with 86 CGG repeats who denied tremor and ataxia. On examination his blood pressure was 125/88 mmHg and his heart rate was 88 bpm. His blood pressure was being controlled with irbesartan and hydrochlorothiazide. His BMI was 26.9. Finger-to-nose touching showed no tremor and his tandem walk was without difficulty. He had slight increased tone on the right and left extremities with symmetrical movement. Deep tendon reflexes were normal, and he scored a 2+ in all four extremities. His vibration sense, tactile sensation, and cold sensation were also normal. No cognitive abnormalities were present on neuropsychological examination and no psychiatric symptoms were reported.

The MRI revealed the MCP sign (Figure 1C). Additional white matter changes were seen in the splenium of the CC.

Case 4 is a 60-year-old right-handed, male carrier with 74 CGG repeats who denied tremor and ataxia. On examination he had a blood pressure of 152/86 mmHG and a heart rate of 89 bpm. He had a BMI of 27.9. His neurological examination showed decreased vibration sense in the lower extremities. Deep tendon reflexes were 1–2+ in the upper and 2+ in the lower extremities. He had no rest or action tremor, gait ataxia, or dystonia. No cognitive abnormalities were present on neuropsychological testing and no psychiatric symptoms were reported.

The MRI showed mild cerebellar volume loss, with MCP sign bilaterally (Figure 1D) and subtle inferior cerebellar white matter changes.

Case 4 visited for follow up examination at age 64, at which time he had developed a head tremor and mild intention tremor. He also demonstrated subtle gait ataxia, taking only three steps on tandem walking. His MRI at age 64 confirmed the previous findings (Figure 1E).

Case 5 is a 61-year-old, right-handed male carrier with 89 CGG repeats, who denied any history of tremor. He noted that he had no history of falling but he experienced some instances of unsteadiness when turning around, possibly attributable to a 4 cm difference in leg lengths secondary to a congenital vascular malformation in one leg that impacted growth. On examination, his blood pressure was 107/68 mmHg and his heart rate was 55 bpm. He had a BMI of 26.5. There was no sign of tremor during finger-to-nose touching. Along with having mild balance problems while turning, he had instability on tandem walking during the first few steps. With practice, he was able to perform at least 6 steps without missteps. He had normal reflexes in his upper extremities and knees, all +2. His right ankle reflex was 1+, and there was no reflex in his left ankle. He had decreased vibration sense bilaterally in both big toes but normal vibration sense at the ankles bilaterally. Additionally, pinprick sensation was slightly decreased in the great toe. No cognitive abnormalities were present on neuropsychological examination and no psychiatric symptoms were reported.

On MRI, the T2 images showed the MCP sign (Figure 1F) and white matter hyperintensity in the splenium of the CC. He also had mild brain atrophy.

## Methods

This study was carried out in accordance with the recommendations of Institutional Review Board at the University of California, Davis with written informed consent from all subjects. All subjects gave written informed consent in accordance with the Declaration of Helsinki. All subjects gave written informed consent for publication of their cases. The protocol was approved by the Institutional Review Board at the University of California, Davis. The study protocols involved a detailed medical history and neurological examination, along with neuropsychological testing, molecular studies of *FMR1*, and MRI. All participants were English-speaking men between the ages of 50 and 70 years, and each was known to be a carrier of the *FMR1* premutation prior to study enrollment.

The neurological examinations were performed by a medical doctor and included cranial nerve function, tone, rigidity, reflexes, sensation, extrapyramidal function, and gait. In all five cases, signs of tremor and ataxia were judged to be absent.

Neuropsychological testing included the Wechsler Adult Intelligence Scale, Wechsler Memory Scale, and Behavioral Dyscontrol Scale. Cases 1, 2, 4, and 5 completed the Wechsler Adult Intelligence Scale, Third Edition (WAIS-III). Case 3 was tested with the Wechsler Adult Intelligence Scale, Fourth Edition (WAIS-IV); however the entire test protocol was not completed. Scores for each case are reported in Table 1.

**Table 1:** Overview of findings for cases 1–5.

	**Case 1**	**Case 2**	**Case 3**	**Case 4**	**Case 5**
Age at exam (years)	65	50	62	60	61
CGG repeat length	84	102	86	74	89
BMI (kg/m^2^)	29.3	25.8	27.0	26.5	26.5
Blood pressure (mmHG)	177/87	147/82	125/88	152/86	107/68
Full scale IQ (WAIS-III)	128	120	N/A^*^	116	116
MRI findings	MCP sign, white matter disease in insula	MCP sign, Arteriovenous malformation	MCP sign, white matter disease in splenium of corpus callosum	MCP sign, mild cerebellar volume loss	MCP sign, white matter disease in splenium of corpus callosum
FXTAS symptoms	None	None	None	None	Slight balance problems, may be attributed to leg length difference

* *This subject completed four subtests of the WAIS-IV (Wechsler Adult Intelligence Scale, Fourth Edition) that are equivalent to the WASI-II administration (Wechsler Abbreviated Scales of Intelligence, Second Edition), which yielded a full scale IQ estimate in the range of 108–118*.

Psychiatric symptoms were evaluated using the Symptom Checklist-90-Revised (SCL-90-R) and the Structured Clinical Interview for DSM-IV (SCID-4).

Genomic DNA was isolated from 3 ml peripheral blood using Gentra Puregene Blood Kit (Qiagen, Valencia, CA) following standard procedure. CGG trinucleotide repeat size was determined using a combination of PCR and Southern blot analysis. PCR was performed using *FMR1* specific primers lying outside the CGG repeat (21). Southern blot was performed by digestion of 7–10 μg of genomic DNA with the restriction enzymes Eco RI and Nru I and the digested DNA was separated on 0.8% agarose gel containing Tris-acetate-EDTA buffer. DNA was then transferred on a nylon membrane and hybridized with the FMR1 -specific genomic probe StB12.3 (22).

Presence of the MCP sign was confirmed by neuroradiological examination of MRI images collected on a Siemens 1.5T or 3T scanner using pulse sequences. In cases 1, 2, 3, and 5, two different pulse sequences were used. The first was a turbo spin echo (TSE) acquired in 48 axial slices of 3 mm thickness (no gap) with FOV of 240 mm^2^, TR of 4,000 ms, TE of 84 ms, and matrix size of 384 × 384. The second pulse sequence was a T2-weighted fluid-attenuated inversion recovery (FLAIR) in which images were acquired in 104 sagittal slices of 1.9 mm thickness (no gap) with FOV 243 mm, 512 Å~ 512 matrix, TR of 5,000 ms, TE of 455 ms, and inversion time 1,700 ms. In case 4, the MRI included 1.5 mm corona spoiled gradient echo (SPGR) and 3 mm axial proton density and T2-weighted scans.

## Discussion

Here we present five cases of male premutation carriers who have white matter disease in the MCP, a classic sign of FXTAS, but do not demonstrate clinically significant tremor or ataxia. Case 5 has noted mild balance problems, but he also had a leg length discrepancy, which may have accounted for this subtle sign. Although some cases have medical problems such as high blood pressure, or decreased vibration sense in the lower extremities typical of mild neuropathy, the major clinical criteria for FXTAS are not present in these cases. This causes a concern regarding their prognosis for FXTAS since the radiological findings show evidence of neurodegeneration but there are no signs of tremor and ataxia; symptoms that would form the basis of the diagnosis of FXTAS (10, 23). The recent report by Wang et al. suggests that there is a continuum of premutation involvement that starts with brain volume loss by middle adulthood (24). This may progress into the neurodegeneration of FXTAS, including white matter disease in the MCP or elsewhere in the brain, such as the splenium of the CC (25, 26). The cases presented here demonstrate that the MCP sign can occur even when other symptoms of FXTAS are not present. In case 4, FXTAS symptoms developed within 4 years after the appearance of the MCP sign.

It is not clear what exactly is the cause of the white matter disease, but the RNA toxicity leading to sequestration of critical proteins for neuronal function, chronic DNA damage repair, mitochondrial dysfunction, dysregulation of iron transport, and the production of the neurotoxic FMRpolyG protein from RAN translation, may all contribute to cell death and white matter disease (2, 27).

Martinez-Cerdeno et al. suggested that the white matter disease in the MCP may be related to microhemorrhages (28). Specifically, they proposed that endothelial cells lining the capillaries become laden with iron and fail to maintain capillary integrity, resulting in the release of erythrocytes that, upon breakdown, release iron. Iron accumulates in the capillaries and white matter tissue, inducing activation of microglia and astrocytes, high oxidative state and/or mitochondrial dysfunction, all leading to myelin loss and axonal degeneration (19, 27, 29). Microhemorrhages may occur more frequently in individuals who have hypertension (30). A large-scale study by Hamlin et al. demonstrated that those with FXTAS are at elevated risk of developing hypertension (31). It is therefore interesting that case 1, case 2, and case 4 had hypertension and in two of these cases this was not adequately treated. Because hypertension is associated with stroke, central nervous system bleeding and white matter lesions, these three cases underscore the need for further research to determine whether there is a similar association between hypertension and the appearance of the MCP sign (32).

Once there is significant white matter disease, the progression of further neurological dysfunction is thought to escalate, although the progression of FXTAS may be quite variable (33). A recent study by Shickman et al. suggested that motor slowing may be measurable in asymptomatic premutation carriers before clinically observed motor or tremor symptoms arise in FXTAS (34). Future studies that examine white matter changes and subclinical motor signs of FXTAS may clarify which carriers are at greatest risk of developing clinical FXTAS symptoms.

A preliminary study demonstrated that the onset of FXTAS correlates with increased CGG repeats, such that the higher the repeat number, the earlier the onset of FXTAS symptoms (35). In the cases presented here, we see both high and medium CGG repeat numbers associated with the presence of the MCP sign with no signs of FXTAS. Genomic studies are needed to decipher if there are other additive deleterious or protective allelic variants or mutations, which can cause early onset of white matter disease or protect against the clinical manifestations of FXTAS.

In the cases reported here, the neurological symptoms of tremor and ataxia are nonexistent so we do not have the clinical findings for a diagnosis of FXTAS. We consider these patients to be at high risk for FXTAS, or pre-FXTAS, because they already have the MRI findings and therefore evidence of neurodegeneration. We have seen a progression of symptoms in Case 4, who had developed FXTAS at his 4-year follow up, and we will continue to follow the remaining four subjects.

## Concluding remarks

The presence of the MCP sign may be more common than originally thought in premutation carriers without neurological symptoms. Their follow-up will guide our recommendations for these carriers in the future. We suspect that avoidance of toxins (such as excessive alcohol, opioids, and inhaled anesthetic agents) and other insults (such as the hypoxia of sleep apnea, or head trauma), in addition to exercise and treatment of medical problems including hypertension or obesity may help to stabilize or slow the progression of FXTAS even in those with white matter disease as reported here (36, 37). Perhaps supplements such as antioxidants, epicatechins or other prophylactic measures may improve mitochondrial function or white matter disease (38, 39). Longitudinal research to uncover the risk and protective factors underlying the development and progression of FXTAS and the development of treatment targets is needed to help these vulnerable individuals with the premutation.

## Data availability statement

The raw data supporting the conclusions of this manuscript will be made available by the authors, without undue reserve, to any qualified researcher.

## Author contributions

JF, FM, YM, JG, FT, DH, SR, VM-C, and RH drafting/revising the manuscript for content. FM formatting the manuscript. FT providing the molecular data. DH, RH, and SR funding study. RH analysis and interpretation of the data.

### Conflict of interest statement

FT one of the topic editors; has received funding from Asuragen, Inc. DH has received consulting fees from Ovid for FXS clinical trial development. RH one of the topic editors; has received funding from Novartis, Marinus, Alcobra and Neuren to carry out trials in Fragile X Syndrome (FXS) and consulted with Zynerba, Fulcrum and Ovid regarding treatment trials in FXS. The remaining authors declare that the research was conducted in the absence of any commercial or financial relationships that could be construed as a potential conflict of interest. The reviewer FN and handling Editor declared their shared affiliation at the time of the review.
